# A Socio-Health Approach to Improve Local Disaster Resilience and Contain Secondary Crises: A Case Study in an Agricultural Community Exposed to Bushfires in Australia

**DOI:** 10.1017/S1049023X22002436

**Published:** 2023-02

**Authors:** Joseph Cuthbertson, Frank Archer, Andy Robertson, Jose Rodriguez-Llanes

**Affiliations:** 1. Monash University Disaster Resilience Initiative, Clayton, Victoria, Australia; 2. Western Australia Department of Health, Perth, Western Australia, Australia; 3. European Commission Joint Research Centre, Ispra Sector, Ispra, Lombardia, Italy

**Keywords:** disaster resilience, health determinants, health security

## Abstract

Recent large-scale disasters have exposed the interconnected nature of modern societies, exacerbating the risk of cascading impacts. Examining elements of community health status, such as social determinants of health, their perceived health status, and how they relate to disaster resilience, can illuminate alternative actions for cost-effective disaster prevention and management. Moreover, agricultural communities are essential to food security and provide a working example of the importance of mitigation in escalation of crises. To that aim, this research examines perceptions of the relationship between disaster resilience and determinants of health, including health status. Participants also reported their views on perceived vulnerable groups in their community and proposed design characteristics of more effective community disaster plans.

Here investigated are these elements in a small agricultural community of Western Australia previously exposed to bushfires. A questionnaire was used based on health elements from the Social Determinants of Health described by the World Health Organization (WHO) and compared this with quantitative data describing the community health status. A mixed methods approach combining qualitative (semi-structured interview) and quantitative (closed questions using a Likert scale) tools was undertaken with a small group of community members.

It was found that community connection and social capital were perceived to provide knowledge and support that enhanced individual disaster risk awareness and preparedness and improved an individual’s disaster resilience. Stress and social exclusion within a community were perceived to decrease an individual’s resilience to disaster. Disaster resilience was reported to be a function of good physical and mental health. To achieve effective disaster planning, community partnership in the development, education, and testing of plans and robust communication were described as essential traits in community emergency plans.

## Introduction

The risk of disasters and their impacts globally continues to increase in frequency and magnitude.^1,2^ An increasingly interconnected world is revealing cascading impacts, some of which were previously hypothesized.^3^ These include, but are not limited to, small but densely distributed events (eg, epidemic outbreaks) escalating into national or transboundary crises^3^ that elicit state responses and above. These responses in turn may affect further sectors in the economy, with consequent social and health impacts. A more integrated and effective approach to prevent and to quickly respond to the threat of hazards becoming disasters is urgently needed.^4,5^ To achieve this, a person-centered focus in prevention and response to crisis has been suggested.^6^ Whilst such an approach is commendable, this focus requires rooting within communities and improved understanding of localized vulnerability profiles and context, which should be used to guide the available capacities and potential solutions to disaster managers, other involved professionals, and the communities themselves.^7,8^

Current evidence suggests that a systematic effort to analyze and manage the root causes of disasters is more effective in reducing disaster risk rather than using traditional management processes.^9^ These include the individual resilience factors of community members. Robust (or lack thereof) determinants of health may influence the outcomes of disaster events upon an individual and/or a community which disproportionally affect vulnerable groups within them.^10^ These groups are often marginalized populations due to gender, age, disability, ethnicity, religion, or sexual orientation, or a combination of these and other characteristics.^11,12^ Comprehensively examining the determinants of health and health status with an impact on disaster resiliency is well-aligned with contemporary frameworks in disaster risk reduction. Whilst disaster practice to date has predominantly focused on emergency management, new thinking proposes that investment in reduction, addressing vulnerability, and improving community capacity and its resiliency provides a greater return on investment.^10^

This paper seeks to address the knowledge gap in how community inhabitants across varying age groups relate disaster risk reduction to their health status and to what extent they perceive drivers of health status as important to being disaster resilient. As a case study to answer these research questions, an investigation was conducted in Dwellingup, a small town in Western Australia, utilizing a questionnaire based on the Social Determinants of Health (as described by the World Health Organization [WHO; Geneva, Switzerland]) and local government data of measured health status.

### Definitions

Vulnerability may refer to physical aspects, such as poor health, versus social and economic vulnerability, like isolation and poverty. The definition of vulnerability in respect to this project is: “*the conditions determined by physical, social, economic, and environmental factors or processes which increase the susceptibility of an individual, a community, assets, or systems to the impacts of hazards.*”^1^ In this context, the definition was applied to describe groups perceived as “vulnerable” in the context of a disaster.

Resilience in this study refers to the capacity of individuals, and in the aggregate communities, to anticipate, absorb, adapt, or recover timely from a shock with minimal perturbation to their basic functions and with possibility for improving them.^12^

### Study Setting

Dwellingup is a town located on the urban rural fringe in a timber and agricultural area of the Darling Range in Western Australia, 97km south of Perth. Its location, within an easy drive of the capital city Perth, makes it an attractive destination for tourists and visitors for its forests, local produce, and scenery. The features of its natural beauty are also linked to its risk, as it is in a heavily wooded region with hilly terrain with a hazard of bushfire. A bushfire in 1961 resulted in vast destruction of land and homes in Dwellingup and the surrounding community. One hundred thirty-two houses were destroyed and 800 people were left homeless.^13^ Dwellingup suffered bushfire again in 2007, resulting in wide-spread property and forest destruction. Whilst Dwellingup is close to the State capital, the terrain reduces access to the town and emergency response capability is dependent primarily on local volunteer support who would rely on external support for response to large emergencies. These characteristics are relevant as they are common to many small regional towns in this State.

## Methods

A mixed methods approach was used in this study. Qualitative and quantitative data were collected through key informant semi-structured interviews. Interviews were guided by means of a questionnaire inspired by the Social Determinants of Health as described by the WHO (Appendix 1; available online only).^14^ Using purposive sampling, 33 face-to-face interviews were requested of local community members. Of these, 18 accepted to be interviewed, while the remaining 15 did not respond to the email request. No further follow-up of non-responders was conducted. No potential interviewees refused to participate once they accepted. Participants were identified using purposive sampling via a local community leader. Participant demographics are presented in Table 1. Data collection was conducted from March 2018 through May 2019, and a typical interview lasted between 45 – 60 minutes. Interviews were ceased when responses indicated no new information was obtained. The questionnaire (Appendix 1) data were examined to identify community perception of vulnerability and importance of health determinants to individual disaster resilience.

**Table 1.** Participant Demographics

Characteristics	No.
***Gender***	
Male	11
Female	7
***Relationship Status***	
Single	4
Partner	14
***Age (years)***	
Below 20	0
21–30	1
31–40	0
41–50	6
51–60	2
Above 60	9

Participants were asked to grade their perceptions of the relevance of determinants described in each question on a scale of one to ten (one being very low; ten being very high). Qualitative analysis of the remaining interview questions used narrative inquiry according to the six-step methodology described by Braun and Clark where a theme “captures something important about the data in relation to the research question and represents some level of patterned response or meaning within the data set.”^15^ The themes were reviewed to identify similarity or overlap, and whether unification of codes into central themes or sub-themes was appropriate. Finally, external quantitative data describing the community health status were collected from the Australian Bureau of Statistics (ABS; Canberra, Australian Capital Territory, Australia).^16^

### Ethical Considerations

All respondents provided written informed consent prior to participation and did not receive any incentives to participate in the study. Ethical approval was requested and obtained from Monash University (Clayton, Victoria, Australia) Human Research Ethics Committee (HREC 7539).

## Results

Thematic analysis of the qualitative data was conducted, consistent with the methodology described by Braun, et al.^15^ Respondent demographics are shown in Table 1 and community demographics are shown in Table 2 and Table 3.

**Table 2.** Dwellingup 2016 Census Demographics^10^

Demography	Outcome
Population	557
Male	51.8%
Female	48.2%
Median age	46
Families	145
All private dwellings	308
Average people per household	2.4

**Table 3.** SEIFA Scores, Murray Western Australia – Wheatbelt^15^

Socio-Economic Indexes for Areas (SEIFA)	Score	Decile
Index of Relative Socio-Economic Advantage and Disadvantage (IRSAD)	1010	6
Index of Relative Socio-Economic Disadvantage (IRSD)	1013	6
Index of Economic Resources (IER)	1049	8
Index of Education and Occupation (IEO)	943	4

The latest census in 2016 recorded a population of 557 in Dwellingup with the following demographics^16^ (Table 2).

The Socio-Economic Indexes for Areas (SEIFA) index for Dwellingup was accessed via the ABS census data to determine the relative advantage and disadvantage of the area compared to the rest of Australia. A SEIFA score is an average using a set of four indexes which provide summary measures derived from the ABS census to understand the relative level of social and economic well-being of people and households within a given region. The definition applied by SEIFA of relative socio-economic disadvantage relates to access of material and social resources, and the ability to participate in society based on characteristics of people, families, and dwellings within that area. The SEIFA measures have been reported as deciles where the lowest scoring 10% of areas are given a decile number of one, up to the highest 10% of areas which are given a decile number of ten. The four SEIFA indices are: the Index of Relative Socio-Economic Disadvantage (IRSD), the Index of Relative Socio-Economic Advantage and Disadvantage (IRSAD), the Index of Economic Resources (IER), and the Index of Education and Occupation (IEO).^17^

The SEIFA scores of the region (Murray) that the town of Dwellingup resides within describe how both Dwellingup and the surrounding area compare relative to Australia. Table 3 shows the Murray SEIFA scores and demonstrates that the area is above average in respect to socio-economic advantage and economic resources (IRSAD, IRSD, IER) and slightly below average in respect to education and occupation (IEO) compared to other Australian regions.

The Australian Institute of Disaster Resilience (AIDR; Melbourne, Victoria, Australia) Initiative has developed the Australian Disaster Resilience Index. The index provides a measure of disaster resilience for selected Australian geographic areas. Index values range between zero and one, where zero is low resilience and one is high. Current reporting does not include the town of Dwellingup, however two towns measured within the same region (Murray) are reported as moderate and low.^18^

### Vulnerability Profile

It was requested for participants to grade various listed and non-mutually exclusive community groups according to their perceptions on vulnerability levels. On average, elderly (8.8), disabled persons (8.8), and visitors (8.5), closely followed by children (8.1), were those perceived as most vulnerable. Locals (3.8) and those unemployed (4.2) were perceived as the least vulnerable.

When asked how important health is in reference to disaster resilience, respondents ranked it with a mean score of 8.5, demonstrating high value of health status. Participants in the study were further questioned on various social determinants and health, and contributions to disaster resilience. Overall, mental health and social exclusion were perceived as most influential and equally important factors for disaster resilience (8.6). Insurance was also highly ranked (8.0), and other noteworthy factors were stress levels (7.8), early life development and education (7.7), and living with chronic disease (7.2). Religion (1.5) was thought to have a very limited contribution to disaster resilience from the respondents.

### Reviewing Potential Themes

**Figure 1.**
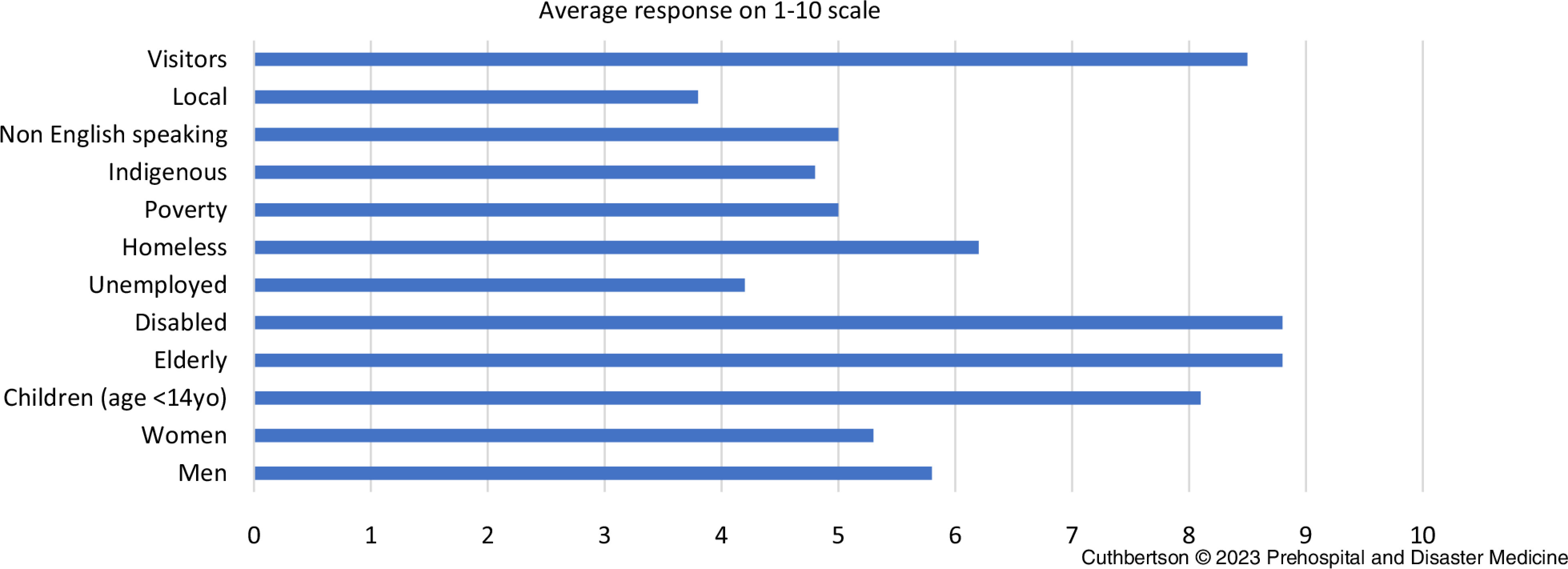
Participant Perceptions of Vulnerable Community Groups.

**Figure 2.**
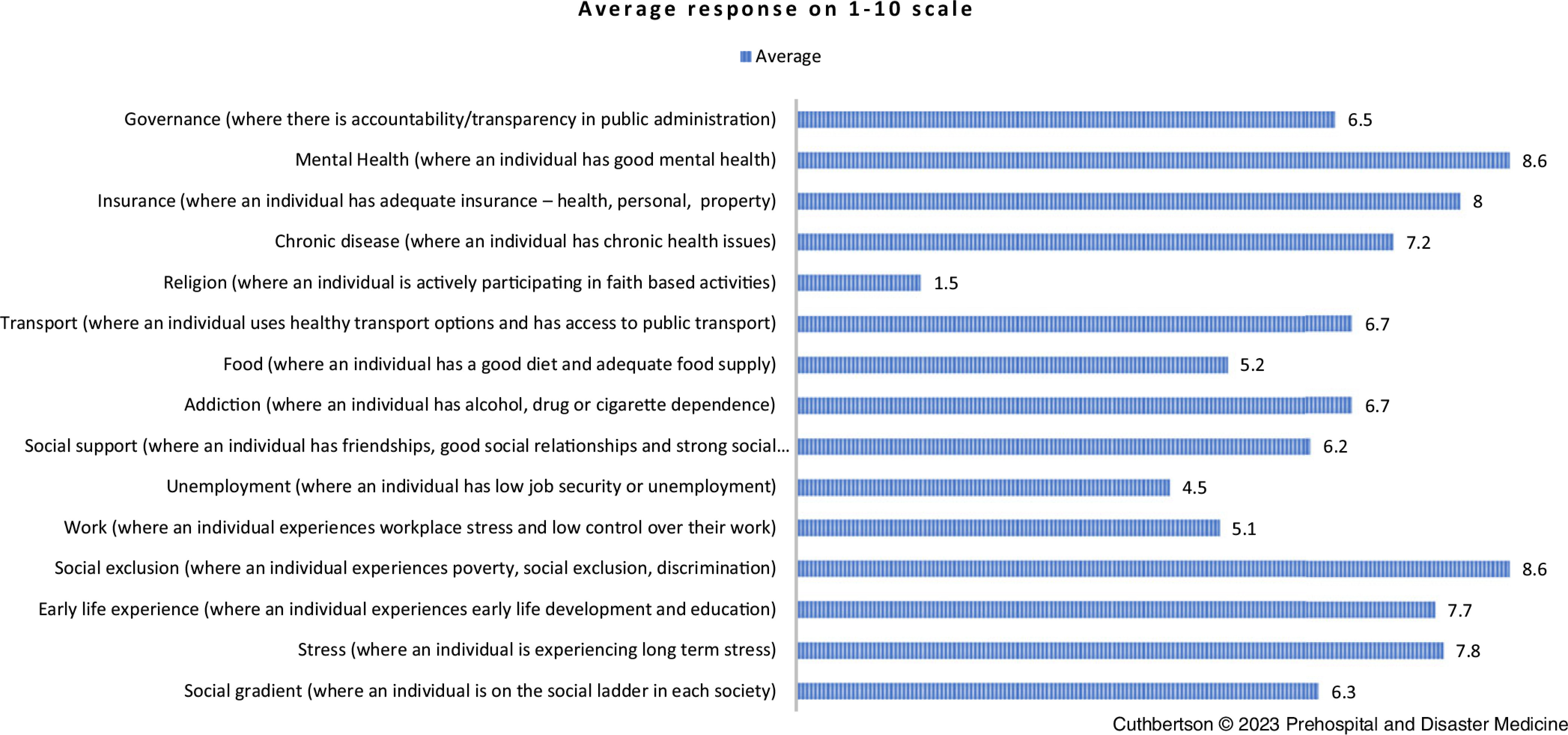
Participant Perception of Social Determinant’s Relevance to an Individual’s Disaster Resilience. Note: A ranking of 0 refers to very low relevance of the particular item and 10 very high; the numbers typed near the end of the bars refer to the calculated mean.

Several themes emerged from findings of the qualitative interview questions. Community connection and sense of belonging as an attribute of disaster resilience was evident in participant responses and constructed Theme 1. Equally, a lack of community connection, social isolation, and/or chronic stress were perceived as increasing vulnerability to disaster; this contextual risk perception was developed as Theme 2. Responses from participants when questioned on vulnerable groups within the community (Figure 1) identified strong beliefs; the findings of this were explored further to create Theme 3. Good physical and mental health were considered to be dependencies of a person’s disaster resilience, however perceptions of the value of health determinants varied (Figure 2). The results of this were unified into Theme 4. A singular theme of robust communication, planning, and community partnership were identified as elements of effective disaster plans and formed Theme 5. The final five themes resulting from this analysis are presented in Box 1.

**Box 1.** Final Five Themes Identified in Study.

1. Local knowledge, sense of community, and participation are enhancers of disaster resilience.
2. Chronic stress and social exclusion and/or lack of connection within a local community increases an individual’s vulnerability to disaster.
3. Perception of disaster vulnerability varies between community demographic groups.
4. Disaster resilience is a function of good physical and mental health.
5. Effective disaster planning requires community partnership and robust resource planning, risks awareness, communication, and coordination in development, training, and testing. Robust communication is an essential trait of disaster plans.

### Theme 1: Local Knowledge, Sense of Community, and Participation are Enhancers of Disaster Resilience

Interviewees demonstrated strong beliefs in respect to local knowledge and community connection as factors that influenced disaster resilience. When describing elements of a disaster resilient community, respondents identified that strong networks within the local community contributed to improved disaster resilience. Underpinning these community relationships was a sense of common bonding and care between community members. Examples provided by interviewees included:

*Common identity (sense of belonging), concern for fellow humans at an individual level. Having a lack of selfishness in respect to helping the community and having investment in the future of the community.*

*Having local knowledge, knowing the history of the place.*

*Knowing neighbors (I didn’t know my neighbors in the suburbs), there’s more community connection in the country.*

Moreover, when this theme was explored further, respondents demonstrated strong feelings of independence and self-reliance as a community:

*The size of a community (sic) results in different meanings as to what a disaster really is. Small communities are more resilient because they feel they are on their own, therefore they won’t wait for help because they don’t think it will come.*

The local community was viewed as a source of knowledge and support that was accessible if an individual was connected to it; however, it was noted that whilst an individual may reside in the community, this did not imply that they were connected to it. This finding was explored further in Theme 3.

### Theme 2: Chronic Stress, Social Exclusion, and Disconnect from the Community Increases Vulnerability to Disaster

Chronic stress (where an individual is experiencing long-term stress) and social exclusion were perceived by many to have a significant impact on an individual’s disaster resilience. When explored further to understand the community member perspective of why they considered stress or social exclusion as having a significant impact on an individual’s vulnerability related to disaster resilience, they described:

*Yes – ability to cope in times of stress and physical capacity to deal with crisis. Mental and physical strength so that you’re not frozen to deal with the stress and that you are physically able to meet the demand. Attachment to the community has a positive and negative context as the investment loss can be profound.*

*Not being a part of the community network increases risk. There are loners that live here that aren’t connected within the community.*

These findings are consistent with research previously recognizing these elements and their relationship to resilience and disaster risk reduction.^4,12^

### Theme 3: Perceptions of Disaster Vulnerability Varies between Community Demographic Groups

Several groups within and external to the community were perceived to be at greater risk by the interview participants. Holiday homeowners, loners within the community, new community residents, and tourists were considered at risk/vulnerable due to their perceived lack of local knowledge of the environment, the risk it posed, required preparedness to mitigate it, and lack of community connection. Key responses that informed this theme included:

*When you have a lack of local knowledge. Tourists (sic) make us vulnerable when they come here and do things that put us at risk. We’re also vulnerable because we need them to support our economy; we just need to educate them when they come here.*

*Yes, elderly as they aren’t physically able to respond to local risks, especially fire. Tourists have a lack of understanding and knowledge of the area and the risks and are less able to assess the risk. Weekenders are not well-engaged with the community and don’t understand the risks here. Children and teenagers are less engaged in local community activities and don’t understand the community structure.*

When the reason for the sense of vulnerability related to these groups was explored, in both cases, the rationale was related to decreased levels of community connection described in Theme 1. In the case of tourists, weekend homeowners, and the elderly, there was a perception of lowered community connection and involvement that decreased these populations risk knowledge and awareness. Coupled with this, the elderly were perceived to be at greater risk due to potential mobility challenges, and both the elderly and children were considered as having greater need for support in times of crisis. Two interviewees who lived in the area but not in town perceived a difference in risk management between rural lot dwellers and those that lived in town.

### Theme 4: Disaster Resilience is a Function of Good Physical and Mental Health

Both physical and mental health were consistently perceived to be of high value in relation to a person’s capacity and resilience. Interestingly, mental health was rated highest in significance related to an individual’s disaster resilience compared to all other health determinants. This finding was also repeated in interview questions related to the participant’s perception on the importance of health in reference to disaster resilience (Question 6):

*Yes, so you have capacity to make decisions and not be bound by infirmity.*

*Yes, because from start to finish, disasters affect your resilience. If you’re fit and healthy (mind and body), you have greater ability to respond and recover and lift yourself up. It’s going to be very important.*

### Theme 5: Effective Disaster Planning Requires Community Partnership and Robust Resource Planning, Risks Awareness, Communication, and Coordination in Development, Training, and Testing – Robust Communication is an Essential Trait of Disaster Plans

Community risk management and risk literacy with the focus on leadership and effective, local risk communication that meets the needs of the community were considered key to describing and operationalizing disaster management plans. A clearly described need for access and provision of reliable, timely information at a recognized meeting location was evident in all interviewee responses. When describing the rationale for this need, interviewees described:

*Having a designated controller/coordinator who is allocated early and that this is known early by all. Decide early on whether to go or to stay, having a preparedness pack if you are going to go, having meeting points for gathering (sic) identified.*

*Who’s in charge, having a hierarchy so there’s no confusion when disaster hits, this keeps everyone together.*

*Getting information out to communities, evacuation plans, knowing who’s responsible for what. Everyone should know the plan, communicate the plan well before.*

## Discussion

The participants of this study reported that social exclusion (where an individual experiences poverty, social exclusion, and/or discrimination) was considered to have a significant impact on an individual’s disaster resilience. Traditional disaster resilience has often been framed by access to resources and physical preparation (ie, food supplies, firefighting equipment) rather than community connection. Respondents to this study expressed value in community participation and connection, identifying the strength in a shared and supported response to disaster. This finding is consistent with research conducted by Norris, et al who found that populations with low socio-economic status are at greater risk of mental health consequences following a disaster, due to feelings of lack of self-worth and income stress.^19^ A previously conducted literature review has identified lack of social support, female gender, prior traumas, resource loss, human loss, and poor physical or mental health as likely indicators of psychological resilience to disasters.^12^ The findings of this study are consistent with this literature review and serve to further exemplify the utilization of Social Determinants of Health as indicators of community disaster resilience.

Community strength and connectedness was a finding by respondents who considered them a factor that enhanced a community member’s resilience. This connectedness was perceived as a strength as it facilitated support between community members. When describing a disaster resilient community, a resident suggested:

*Capacity to bind people together to help each other for protection at the time and then help each other to return to normal as soon as possible.*

Social connectedness has been previously explored by Lacoviello, et al in reference to the impacts of disaster. Their findings showed that supportive social networks increase an individual’s resilience, and importantly, enhancement of them pre-disaster impact had a positive effect on mitigating psychological trauma post-event.^20^ Further to this, Aldrich has also reported on the critical role of social capital and networks in disaster survival and recovery.^21^

A low indication of the relationship between faith and resilience may be reflective of the secular nature of Australian society^22^ or the localized nature and sample of this study. When compared to research in other areas of Oceania, faith and religion have been found to be a common factor for resilience amongst survivors of tsunami.^23^ Further to this, other Western societies have found that older adult survivors reported faith and religious practice as coping mechanisms following a disaster.^24^

Provision of information featured strongly in this research. This is consistent with the research findings of Norris, et al who describe elements of adaptive capacities of communities affected by disaster.^19^ These authors also identified that the lack of information created community stress, a finding consistent with a respondent in this research who reported that:

*Being by yourself with no resources and without knowledge on what to do makes you vulnerable. A lack of information in the 2009 fires meant people were reacting to rumors and whispers of what was happening. You need a central point of communication, somewhere where the community can receive information and ask questions. Without this, the community splits apart.*

This is consistent with previous research identifying that maintaining trust and mitigating fracturing of communities during and after disasters is achieved by timely, factual communication from leadership.^25,26^

The results of socio-economic status derived from Australian Government data for Dwellingup and the surrounding area identified that the area is above average in respect to socio-economic advantage and economic resources and slightly below average in respect to education and occupation. This would suggest that the inhabitants of the Dwellingup area do not show significant socio-economic advantage or disadvantage in comparison to other Australian regions. This finding is of value when considering the community questionnaire results and whether bias due to underlying advantage or disadvantage is pre-existing.

A gap in understanding community resilience is related to the identification and measurement of vulnerable communities. Garlick reviewed efforts by the Victorian Government to address identification of vulnerability following recommendations of the 2009 Victorian Bushfires Royal Commission (Victoria, Australia). In this review, Garlick describes policy actions taken to reduce the commission’s definition of vulnerability and thereby the scope of action required and consequently undertaken.^27^ Whilst the author acknowledges that the initial scope as described by the Commission was unmanageable, the actions subsequently taken demonstrate a lack in capacity to adequately address this complex issue. In particular, the practice of shared responsibility, noted in the National Strategy for Disaster Resilience, was reported missing in terms of sector and department collaboration. Vulnerability arises from social, cultural, health, and environmental interactions; consequently, no single agency is equipped to assume to adequately respond to identified needs.^9^ These groups, however, are inevitably best placed to assess their own needs and to plan how to meet them during and after emergencies.

Significant barriers in accessing basic needs by older persons/the elderly have also been reported, which can exacerbate challenges faced by older persons in preparing for and responding to disaster.^24^ The interview respondents in this project consistently reported higher vulnerability of elderly residents in the community to disasters. Recent research investigating the impacts of Hurricane Katrina (2005; Gulf Coast, USA) on older adults also found differences in risks in disasters compared to the community they reside in. Identified challenges included physical and psychological health barriers and the inability to evacuate without assistance in preparation, transportation, and pet care.^24^ As noted by an interviewee:

*Elderly, due to lack of mobility, they have a greater dependency and need for transport and can become disconnected from the community.*

This report contributes to the evolving research base investigating older persons in disasters.^28–30^

The WHO Risk Reduction and Emergency Preparedness Strategy for the health sector and community capacity development reflects the recommendations of a global consultation organized by the health action in crisis cluster. This strategic framework signals a shift from a traditional, short-term focused emergency management doctrine to one of capacity building, developing resilience, and reducing vulnerability. The challenge in achieving this goal, as described by the strategic framework, is “establishing systematic capacities, such as legislation, plans, coordination mechanisms, and procedures, institutional mechanisms and budgets, skilled personnel, information and public awareness, and participation that can measurably reduce future risks and losses.” This strategy recognizes the importance of applying a “whole of health” approach and utilizes the WHO definition of health as the benchmark for intervention effectiveness. This strategic direction complements efforts in other areas, notably the agenda for Sustainable Development, and the Sendai Framework for action.^31,32^

The Australian Business Roundtable for Disaster Resilience and Safer Communities report found that to build greater resilience to disasters in the States and Territories, the government should mainstream and embed resilience across all aspects of policy and decision making, prioritize resilience investments by considering their broader economic and social benefits, improve understanding of disaster risks, costs to society, and resilience building activities to improve resilience, and collaborate and coordinate to build resilience and address the long-term costs of disasters.^33^

The findings of this study are reflective of key international and national recommendations for strengthening community resilience and recognizing health and social factors as actionable determinants of disaster resilience and vulnerability reduction.

## Recommendations

The following are suggested recommendations:

1. Physical and mental health and well-being were viewed by the community as fundamental to a person’s disaster resilience. When planning is undertaken to improve a community’s disaster preparedness, the health status of the community should be assessed and incorporated into planning and programs. Improving the health status of a community is a policy with many benefits, including disaster resilience building.
2. Social inclusion (or lack thereof) was noted as a driver of resilience to disaster. Disaster plans and programs require actions to identify isolated community individuals pre-impact, follow them adequately, and seek to engage these groups in local disaster risk reduction.
3. Communication and community participation in disaster risk reduction planning enhances engagement, knowledge, and local ownership of activities to increase resilience. Planning and programs should be framed with a community participatory lens to achieve this outcome.

## Limitations

This study sought to understand how the Social Determinants of Health inform local disaster resilience. This study is limited by small numbers (n = 18) of participants and a lack of representation across all social demographic groups within the community. Whilst the community members interviewed live in an area with a well-known pre-existing disaster risk, it is not established how many may have experienced disaster impact. Questionnaire answers may be positively or negatively biased based on the respondents’ personal values and view of the determinant in question.

Whilst SEIFA indices provide information of socio-economic advantage and disadvantage in a given area, their design was not developed relative to disaster risk reduction and/or vulnerability specific to emergency management planning or practice. Furthermore, SEIFA represents an average of all people living in an area and does not capture individual situations of people. Larger areas are more likely to have greater diversity of people and households.^34^

## Conclusion

This study aimed to investigate how the Social Determinants of Health inform local disaster resilience and how characteristics of population health relate to and impact upon disaster risk and vulnerability. Key findings of this study suggest that strong social connection within the community was felt to provide knowledge and support that enhances disaster risk awareness and improves an individual’s disaster resilience. Conversely, stress and social exclusion from the community was perceived to increase an individual’s vulnerability to disaster. Disaster resilience was considered to be a function of good physical and mental health, and effective disaster planning required community partnership in the development, education, and testing, with robust communication as an essential trait of communication plans.

## Supplementary material

For supplementary material accompanying this paper visit https://doi.org/10.1017/S1049023X22002436. click here to view supplementary material
