# Health Status and Disaster Resilience. A Socio-Health Approach to Improve Local Disaster Resilience and Contain Secondary Crises: A Case Study in an Agricultural Community Exposed to Bushfires in Australia – CORRIGENDUM

**DOI:** 10.1017/S1049023X23000158

**Published:** 2023-04

**Authors:** Joseph Cuthbertson, Frank Archer, Andy Robertson, Jose Rodriguez-Llanes

In the original publication of this article, authors Frank Archer and Jose Rodriguez-Llanes were listed with incorrect post-nominals. This has since been corrected in the original article.

## References

[ref1] Cuthbertson, J. , Archer, F. , Robertson, A. , & Rodriguez-Llanes, J. (2023). A Socio-Health Approach to Improve Local Disaster Resilience and Contain Secondary Crises: A Case Study in an Agricultural Community Exposed to Bushfires in Australia. Prehospital and Disaster Medicine, 38(1), 3–10. doi: 10.1017/S1049023X22002436 PMC9885428 36606323

