# Supplementary material for: A Socio-Health Approach to Improve Local Disaster Resilience and Contain Secondary Crises: A Case Study in an Agricultural Community Exposed to Bushfires in Australia
Source: Prehosp Disaster Med. 2023 Jan 6;38(1):3–10. doi: 10.1017/S1049023X22002436 (PMC9885428; doi:10.1017/S1049023X22002436)
Supplement: Supplementary file 1 [file S1049023X22002436sup001.docx]

**Appendix 1. Questionnaire**

1. What are the elements of a disaster resilient community?
2. Describe vulnerability
3. Are some groups in your community more vulnerable to disasters than others? Who? Why?
4. On a scale of 1 to 10 describe how vulnerable the following groups are:
   1. Men
   2. Women
   3. Children (age <14yo)
   4. Elderly
   5. Disabled
   6. Unemployed
   7. Homeless
   8. Poverty
   9. Indigenous
   10. Non English speaking
   11. Local
   12. Visitors
5. Do you perceive other vulnerable groups?
6. Is health important in the setting of disaster? Why?
7. On a scale of 1 to 10 (1: very low, 10: very high), how important is health in reference to individual? disaster resilience:
8. What does it mean to be healthy?
9. When assessing needs of community at risk or affected by disaster what are the top priorities?
10. When disaster plans for communities are developed what should they provide, describe or plan for?
11. On a scale of 1 to 10, rate whether the following traits (based on the WHO definition of SDofH) impact on an individual’s disaster resilience (1: very low impact, 10: very high impact):
    - Social gradient (where an individual is on the social ladder in each society)
    - Stress (where an individual is experiencing long term stress)
    - Early life experience (where an individual experiences early life development and education)
    - Social exclusion (where an individual experiences poverty, social exclusion, discrimination)
    - Work (where an individual experiences workplace stress and low control over their work)
    - Unemployment (where an individual has low job security or unemployment)
    - Social support (where an individual has friendships, good social relationships and strong social networks)
    - Addiction (where an individual has alcohol, drug or cigarette dependence)
    - Food (where an individual has a good diet and adequate food supply)
    - Transport (where an individual uses healthy transport options and has access to public transport)
    - Religion (where an individual is actively participating in faith based activities)
    - Chronic disease (where an individual has chronic health issues)
    - Insurance (where an individual has adequate insurance – health, personal, property)
    - Mental Health (where an individual has good mental health)
    - Governance (where there is accountability/transparency in public administration)
12. Are you male / female (Please circle as appropriate)
13. Please indicate your age with a tick next to the corresponding figures on the scale below.
    - Below 20
    - 21 – 30
    - 41 – 50
    - 51 – 60
    - Above 60
14. Marital status.
    - single
    - relationship
